# Falcon genomics in the context of conservation, speciation, and human culture

**DOI:** 10.1002/ece3.5864

**Published:** 2019-12-07

**Authors:** Justin J. S. Wilcox, Stéphane Boissinot, Youssef Idaghdour

**Affiliations:** ^1^ Center for Genomics & Systems Biology New York University Abu Dhabi Abu Dhabi United Arab Emirates; ^2^ Program in Biology New York University Abu Dhabi Abu Dhabi United Arab Emirates

**Keywords:** captive breeding, falcon (*Falco*), falconry, genomics, hybridization, nonmodel organism

## Abstract

Here, we review the diversity, evolutionary history, and genomics of falcons in the context of their conservation and interactions with humans, and provide a perspective on how new genomic approaches may be applied to expand our knowledge of these topics. For millennia, humans and falcons (genus *Falco*) have developed unique relationships through falconry, religious rituals, conservation efforts, and human lifestyle transitions. From an evolutionary perspective, falcons remain an enigma. Having experienced several recent radiations, they have reached an unparalleled and almost global distribution, with an intrageneric species richness that is roughly an order of magnitude higher than typical within their family (Falconidae) and across other birds (Phylum: Aves). This diversity has evolved in the context of unusual genomic architecture that includes unique chromosomal rearrangements, relatively low chromosome counts, extremely low microdeletion rates, and high levels of nuclear mitochondrial DNA segments (NUMTs). These genomic peculiarities combine with high levels of ecological and organismal diversity and a legacy of human interactions to make falcons obvious candidates for evolutionary studies, providing unique research opportunities in common topics, including chromosomal evolution, the mechanics of speciation, local adaptation, domestication, and urban adaptation.

## Graphical abstract

Here, we review the ecology, evolutionary history, and genomics of falcons in the context of their interactions with humans, and provide perspective on how new genomic approaches may be applied to the study of these topics with implications for several fields of study, including chromosomal evolution, the mechanics of speciation, population structure, domestication, and urban adaptation.

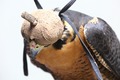

## 1 INTRODUCTION

### 1.1 The cultural, economic, and biological importance of falcons

Throughout history and across the world, few animals have attained the cultural, political, and economic importance of falcons (genus *Falco*). From the early Bronze‐Age culture of ancient Egypt (Porter, 2011; Wilkinson, 2010), to the medieval courts of Europe and Asia (Jaques & Dobney, 2002; Müller, 1993), to the modern monarchies of the Middle East (Wakefield, 2012), falcons have been symbols of power, sovereignty, and regality since the beginnings of recorded history (Negro, 2018). This history likely predates but is closely intertwined with the ancient practice of falconry. Potentially practiced for millennia (Collon, 1983), a sport of nobility for centuries (Jaques & Dobney, 2002; Müller, 1993), and recognized by UNESCO in 2016 as an intangible “cultural heritage of humanity” spread across at least 18 countries (UNESCO, 2016), falconry represents an enduring bond between humans and animals. This bond persists to this day, where it has emerged as an international sport of significant commercial importance. The recurrent President Cup Falcon Competition held in Abu Dhabi awards millions of dollars in prizes and has, in conjunction with several other smaller competitions, helped transform falcons into valuable commodities (Holden, 2018; Jacobs, 2019). Thousands of falcons are traded internationally each year (CITES Trade Database, 2019), and individual falcons are often sold for several thousands of dollars (Fleming, Douse, & Williams, 2011). This trade and the long history of falconry are intermingled with a complex past of direct conflicts between humans and falcons: Many falcon populations were threatened by hunting in the 18th century and 19th century, whereas many falcon populations were decimated in the mid‐20th century as a result of DDT and other organochloride pesticides (Bagyura et al., 2012; Donlan et al., 2006; Holroyd & Bird, 2012). These threats were remedied in part with some of the first massive and successful conservation‐oriented captive breeding programs, which helped restore falcon populations to many wild areas. The breadth, complexity, and endurance of these human–falcon interactions make them emblematic of the present era of ever‐increasing human impacts on ecological systems, the “Anthropocene,” and falcons can provide key insights into extinction, adaptation, acclimation, and resilience in the face of accelerating anthropogenic alterations to the global ecosystem.

While cultural links between the falcons and humans predate recorded history (Negro, 2018), the evolutionary links extend back further still. Under ecological pressures emerging from the same geological trends, falcons diverged and diversified on a timescale similar to that of early hominids (Fuchs, Johnson, & Mindell, 2015). In this short evolutionary time, falcons have undergone several radiations to reach a level of diversity exceeding that of most other genera of birds (Gill & Donsker, 2019). This rapid and recent diversification of *Falco* preserves evolutionary insights into the ecological and geological factors that have driven it, and provides an opportunity for the study of the mechanisms of speciation at several stages. Advances in genomics provide the key for capitalizing on this opportunity, by elucidating at the molecular level the evolutionary forces that gave rise to the diversity of falcons.

## 2 THE BIOLOGY OF FALCONS

### 2.1 Falcon diversity

Falcons belong to the family Falconidae, which contains 7–9 other genera (Fuchs et al., 2015). While all members of Falconidae are birds of prey, Falconidae are not closely related to other raptors (such as eagles and owls) and instead retain song birds and parrots as their closest extant relatives from which they are estimated to have diverged approximately 60 million years ago (Hackett et al., 2008; Jarvis et al., 2014; Prum et al., 2015; Suh et al., 2011; Zhan et al., 2013). This grouping of passerines, parrots, and falcons has been designated Eufalconimorphae (Suh et al., 2011). While there are ongoing debates about the exact number of species contained within it, the genus *Falco* encompasses about 2/3 of all species within Falconidae and contains approximately 40 extant species (Table 1)—a species richness 8–9 times higher than the 4.7 species to a genus average for birds (Gill & Donsker, 2019). Exact estimates of species numbers vary based on classifications of specific lineages into species and subspecies. The IUCN Red List currently recognizes 39 species of falcons (IUCN Red List of Threatened Species, 2018), and this assessment is consistent with traditional phylogenies and reference books (White et al., 1994). Controversy exists, however, around the placement of the barbary falcon, which these prevailing sources categorize as a subspecies of peregrine falcons (*Falco peregrinus pelegrinoides*) despite suggestions of its recognition as an independent species (*Falco pelegrinoides*) on the basis of molecular (Fuchs et al., 2015), morphological, and behavioral data. Merlins (*Falco columbarius*) have also been treated as a single species but molecular data have likewise called this into doubt by highlighting substantial divergence between Eurasian (proposed species: *Falco aesalon*) and North American lineages (Fuchs et al., 2015; Wink et al., 1998). Given the large geographic ranges and local variation observed within many falcon species, controversies abound in the realm of falcon subspecies as well, as exemplified by the synonymizing and recent desynonymizing of the western (*Falco naumanni naumanni*) and eastern (*Falco naumanni pekinensis*) lesser kestrel (Corso, Starnini, Viganò, & Jansen, 2015).

**Table 1:** List of falcon species

Falcon species	Common name	Breeding range	Clade
*Falco ardosiaceus*	Grey Kestrel	Africa	*Dissodectes* Group
*Falco dickinsoni*	Dickinson's Kestrel	Africa	*Dissodectes* Group
*Falco concolor*	Sooty Falcon	Northeast Africa and Middle East	Hobby Group
*Falco cuvierii*	African Hobby	Africa	Hobby Group
*Falco eleonorae*	Eleonora's Falcon	Mediterranean Basin	Hobby Group
*Falco longipennis*	Australian Hobby	Australia	Hobby Group
*Falco severus*	Oriental Hobby	Indian Subcontinent to Indochina	Hobby Group
*Falco subbuteo*	Eurasian Hobby	Africa, Europa, and Asia	Hobby Group
*Falco biarmicus*	Lanner Falcon	Africa, Southeast Europe, Southwest Asia	Large and Mid‐sized Falcons
*Falco cherrug*	Saker Falcon	Eastern Europe and Central Asia	Large and Mid‐sized Falcons
*Falco chicquera*	Red‐necked Falcons	Africa and Indian Subcontinent	Large and Mid‐sized Falcons
*Falco fasciinucha*	Taita Falcon	East Africa	Large and Mid‐sized Falcons
*Falco hypoleucos*	Grey Falcon	Australia	Large and Mid‐sized Falcons
*Falco jugger*	Laggar Falcon	Indian Subcontinent	Large and Mid‐sized Falcons
*Falco mexicanus*	Prairie Falcon	Western North America	Large and Mid‐sized Falcons
*Falco pelegrinoides* a	Barbary Falcon	Northern Africa, Middle East, Central Asia	Large and Mid‐sized Falcons
*Falco peregrinus*	Peregrine Falcon	Global (except Antarctica)	Large and Mid‐sized Falcons
*Falco rusticolus*	Gyrfalcon	Arctic (North America, Europe, and Asia)	Large and Mid‐sized Falcons
*Falco subniger*	Black Falcon	Australia	Large and Mid‐sized Falcons
*Falco aesalon* b	Eurasian Merlin	Northern Europe to Central Asia	Merlin
*Falco columbarius* b	American Merlin	North and South America	Merlin
*Falco sparverius*	American Kestrel	North and South America	[Ungrouped]
*Falco alop*ex	Fox Kestrel	Africa	Old World Kestrels
*Falco araea*	Seychelles Kestrel	Seychelles Islands (Indian Ocean)	Old World Kestrels
*Falco cenchroides*	Nankeen Kestrel	Australia and New Guinea	Old World Kestrels
*Falco moluccensis*	Spotted Kestrel	Indonesia	Old World Kestrels
*Falco naumanni*	Lesser Kestrel	Mediterranean Basin, Central and East Asia	Old World Kestrels
*Falco newtoni*	Malagasy Kestrel	Madagascar and Aldabra	Old World Kestrels
*Falco punctatus*	Mauritius Kestrel	Mauritius	Old World Kestrels
*Falco rupicoloides*	Greater Kestrel	Africa	Old World Kestrels
*Falco rupicolus*	Rock Kestrel	Africa	Old World Kestrels
*Falco tinnunculus*	Common Kestrel	Europa, Asia, Africa	Old World Kestrels
*Falco zoniventris*	Banded Kestrel	Madagascar	Old World Kestrels
*Falco amurensis*	Amur Falcon	Siberia and Northern China	Red‐footed Group
*Falco vespertinus*	Red‐footed Falcon	Eastern Europe and Central Asia	Red‐footed Group
*Falco deiroleucus*	Orange‐breasted Falcon	Mexico to Northern South America	Southern American Group
*Falco rufigularis*	Bat Falcon	Mexico to Northern South America	Southern American Group
*Falco femoralis*	Aplomado Falcon	Mexico to South America	Southern Group
*Falco novaeseelandiae*	New Zealand Falcon	New Zealand (primarily South Island)	Southern Group
*Falco berigora*	Brown Falcon	Australia and New Guinea	[Ungrouped]

a Disputed species sometimes listed as subspecies of peregrine.

b Merlins are sometimes treated as a single species as *Falco columbarius*. For a discussion of divergence between American and Eurasian merlins, see Wink et al. (1998) and Fuchs et al. (2015).

**Figure 1:**
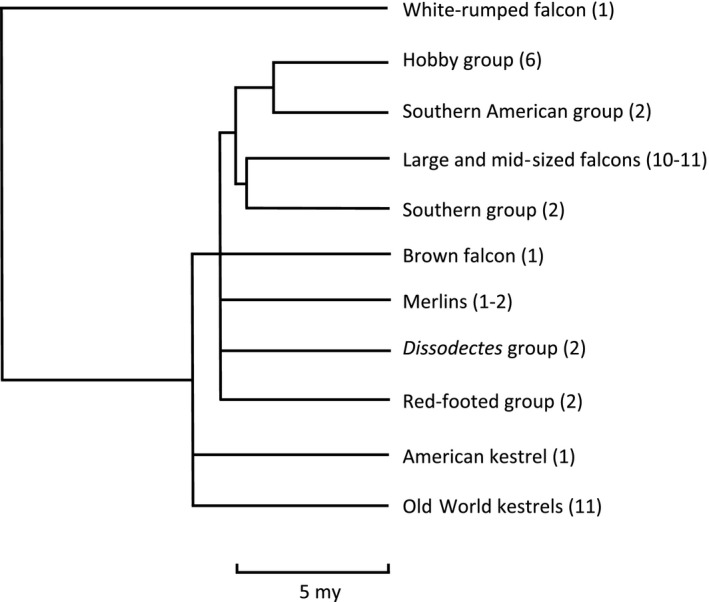
Major groups of falcons with number of species shown in parentheses. The white‐rumped falcon (*Poliheirax insignis*) is shown as the closest living relative of the genus *Falco*. The phylogeny is adapted from Fuchs et al. (2015). It is based on seven nuclear loci and one mitochondrial haplotype sequences, and two fossil calibration points corresponding to splits between subfamilies of Falconidae, and scaled by estimated divergence time

At the intrageneric level, falcons can be broadly divided into three large monophyletic groups (Fuchs et al., 2015): the hobbies (subgenus *Hypotriorchis*), which include six species; the Old World kestrels consisting of 11 species; and finally, 10 or 11 species of large and mid‐sized falcons consisting of the peregrine falcons, the subgenus *Hierofalco*, and close relatives of these (Figure 1). Several other smaller groups of falcon species fall outside of these groups: 1–2 species of merlins, two atypical African kestrels (*Dissodectes*), two Southern American falcons, the single New World Kestrel and two unplaced clades (“Red‐Footed Group” and “Southern Group”) each of which contains two species. The phylogenetic divisions within the falcons reflect important ecological differences. Hunting strategies are generally conserved within, and vary between, the large clades of falcons. Hobbies consist of small and swift aerial predators that tend to take small birds and insects in flight (Bijlsma & Brink, 2005; Parr, 1985; Probst, Nemeschkal, McGrady, Tucakov, & Szép, 2011; Rosen, Hedenström, Badami, Spina, & Åkesson, 1999). Kestrels, however, tend to hover or perch over grasslands and take small vertebrates from the ground (Bildstein & Collopy, 1987; Tella, Forero, Hiraldo, & Donázar, 1998). Among the large and mid‐sized falcons, peregrine falcons specialize on other birds, which they usually take with swoops and dives in flight (Cresswell, 1996; Dekker, 1988; Jenkins, 2000; Tucker, Tucker, Akers, & Enderson, 2000), whereas *Hierofalco* and other member of the clade tend to consume a wider variety of prey that are either taken on the ground or in level‐pursuit aerial flights (Haak, 1982; Poole & Boag, 1988; White & Weeden, 1966).

All extant falcon diversity is estimated to have arisen within the last 5–7 million years and most diversity within subgroups appearing much more recently (Fuchs et al., 2015). Divergence time estimates within the *Hierofalco* are extremely recent and occurred within the last several hundred‐thousand years. These falcons can still hybridize with one another without apparent loss of fertility and can also hybridize with the only slightly more divergent peregrine falcons with reductions in fertility that are more pronounced in females in accordance with Haldane's Rule (Eastham & Nicholls, 2005). More distant hybridization between falcon species is also possible, for example, between peregrines and merlins (termed “perlins”). Polymorphisms in nuclear and mitochondrial genomes are commonly shared between closely related falcon species (Fuchs et al., 2015; Nittinger, Gamauf, Pinsker, Wink, & Haring, 2007; White, Sonsthagen, Sage, Anderson, & Talbot, 2013). These are at least partially the result of incomplete lineage sorting, but could also be due to natural and artificial gene flow between falcon species (Fleming et al., 2011; Nittinger et al., 2007).

### 2.2 The known genome of falcons

At present, genomes have been produced for five falcon species. The saker (*Falco cherrug*) and peregrine (*F. peregrinus*) falcon genomes were sequenced at high depth (>100) using Illumina paired‐end short reads with short and large insert libraries and achieved relatively large scaffold sizes of 4.15 and 3.89 Mb, respectively (Zhan et al., 2013). The common kestrel (*Falco tinnunculus*) genome was sequenced more recently using similar methods, but with a very high depth of approximately 500× and achieved a scaffold N50 of 21 Mb (Cho et al., 2019). Less complete and lower coverage draft genomes have also recently been produced for two additional falcon species: A gyrfalcon (*Falco rusticolus*) draft genome has been assembled with a scaffold N50 of 32.8 kb (Joseph et al., 2018), and a prairie falcon (*Falco mexicanus*) draft genome has been assembled with a scaffold N50 of 3.7 kb (Doyle et al., 2018). As in most birds, the sizes of falcon genomes are relatively small (Kapusta, Suh, & Feschotte, 2017; Zhang et al., 2014), and the assemblies of the genomes of the five falcon species sequenced to date are consistent with genome size of ~1.2 Gb. However, many basic traits of falcon genomes appear less typical. The falcon genomes have more genes annotated than many other avian genomes: Combined homology and de novo transcriptomic‐based approaches suggested between 16,204 and 16,481 protein‐coding genes across the annotated falcon genomes (Cho et al., 2019; Doyle et al., 2018; Zhan et al., 2013). In comparison, a standardized meta‐analysis found an average of 15,101 annotated protein‐coding genes in other birds and placed the peregrine falcon in the top quartile of annotated‐gene counts for birds with 16,242 reference‐based annotations (Zhang et al., 2014). The annotated genes of the peregrine falcon genome are also relatively long: Total gene‐sequence lengths, coding‐sequence lengths, exon lengths, and intergenic distances were all in the upper quartile range for bird genomes, and intron lengths are also above the median length for birds (Zhang et al., 2014). Despite these greater sequence lengths, repetitive and transposable DNA elements are reported to account for a smaller portion of the falcon genome than typical of birds (Cho et al., 2019; Zhan et al., 2013; Zhang et al., 2014). Instead, a lack of deletions might explain some of the trends toward greater sequence lengths: An analysis of genome size evolution in both birds and mammals reported the peregrine falcon as having the lowest rate of microdeletions (i.e., deletions <30 bp) of the seven birds that were compared Kapusta et al., 2017).

Falcon genomes have experienced a high number of mitochondrial DNA insertion events into their nuclear genome, as evidenced by *nu*clear *m*i*t*ochondrial DNA *s*egment (NUMT) counts that are in the upped quartile among birds (Liang, Wang, Li, Kimball, & Braun, 2018). These NUMTs account for approximately 49kb of the total falcon genome (Nacer & do Amaral, 2017). These findings, based on the peregrine and saker falcon genomes, suggest that the numbers of NUMTs in falcons are 2–3× times higher than numbers reported from chickens and exceed reports from many other vertebrates, including those of mammals with much larger genomes. While most NUMTs are small, more than 90% of the peregrine and saker falcon mitochondrial genome is represented by insertions into their nuclear genome and one particularly long insertion representing >70% of the total mitochondrial genome can be found in both sequenced falcon species; this insertion represents 32 gene sequences (including those for tRNAs) the control region and a minisatellite, although all protein‐coding genes were reported to contain at least one aberrant stop codon. NUMT insertion rates vary by two orders of magnitude in birds (Liang et al., 2018), and the reasons for the relatively high numbers of mitochondrial insertions in falcons are unclear, but phylogenetic analyses suggest that many regions of the mitogenome have independently inserted into the falcon genome. As such, these insertions may preserve a record of ancestral states within the falcon mitogenome (Nacer & do Amaral, 2017).

At present, complete mitogenomes are available for nine species of falcons: the American kestrel (Gibb, Kardailsky, Kimball, Braun, & Penny, 2006), peregrine falcon (Ryu, Lee, & Hwang, 2012); merlin (Dou et al., 2016); saker falcon (Lu, Lu, Li, & Jiang, 2016); lesser kestrel (Wang et al., 2016); gyrfalcon (Sveinsdóttir, Guðmundsdóttir, & Magnússon, 2017); prairie falcon (Doyle et al., 2018); amur falcon (Yang, Yang, Wang, Lu, & Li, 2018); and common kestrel (Unpublished, see NCBI Accession: EU196361). While these mitogenomes demonstrate similar patterns of organization to other raptors, they do contain a few intriguing features related to their control regions (Lu et al., 2016; Ryu et al., 2012; Sveinsdóttir et al., 2017; Yang et al., 2018). First, their control regions contain two minisatellites that differ in repeat length and motifs between species and which show intraspecific variation in number of repeats within some species—one of these minisatellites also appears to have been lost in the amur falcon and merlin. The second is the presence of a duplication and degeneration of the entire control region that appears to have fixed across *Falco* species. Similar duplications have been reported as evolutionary parallelism in other birds and squamates and have been suggested to have functional potential (Skujina, McMahon, Lenis, Gkoutos, & Hegarty, 2016). Mitochondrial heteroplasmy has also been reported in some falcons (Fuchs et al., 2015), although its prevalence and significance have not been assessed. The many NUMTs reported in falcons should preserve a record of past mitochondrial states and could make them excellent candidates for exploring the evolutionary history of mitochondrial variations. On the other hand, the ubiquity of NUMTs in falcons may complicate other analyses relating to mitochondrial DNA, particularly as many GenBank sequences attributed to mtDNA have since been identified as having nuclear DNA origins. Mitochondrial barcoding in falcons should also be conducted with caution: While mitochondrial and partial‐mitochondrial haplotype sequences do appear to be effective at distinguishing between some closely related species of falcons (Fuchs et al., 2015; White, Sonsthagen, et al., 2013), studies on peregrine falcons suggest that recent expansions and incomplete lineage sorting make mitochondrial haplotypes ineffective for identifying subspecies (White, Sonsthagen, et al., 2013). Insertions of avian NUMTs have, on the other hand, been shown to generally lack homoplasy and may therefore be particularly phylogenetically informative in the context of genomic studies (Liang et al., 2018).

### 2.3 Chromosomal organization and arrangements in falcons

The falcon genomes are supported by detailed karyotypes across ten species. Interestingly, falcons are one of the few bird lineages deviating from the typical 2N ~ 80 conserved chromosomal number seen across birds (Joseph, 2017). With a 2N range of 40–52, falcons have atypically low chromosome counts. However, like other birds, falcons do show substantial variation in chromosome size, allowing chromosomes to be classified as either macrochromosomes or microchromosomes. As a general trend across diapsids (Lepidosaurs and Archosaurs), microchromosomes are concentrated closer to the center of the nucleus during interphase and are characterized by disproportionately high concentrations of genes, higher recombination rates, and a higher GC content accompanied by more CpG islands (Joseph, 2017). However, the extent to which these properties arise from physical aspects of microchromosomes themselves, or arise from aspects of the DNA sequences that occur on microchromosomes, remains unresolved (O'Connor et al., 2019). Lower chromosome counts in falcons are driven by fusions of microchromosomes into macrochromosomes; whereas most birds possess ~10 pairs of macrochromosomes and ~30 pairs of microchromosomes, falcons possess 7–11 pairs of macrochromosomes and 13–16 pairs of microchromosomes. It is unclear whether former microchromosomal regions of the falcon genome that have fused to macrochromosomes retain the properties of microchromosomes—as expected if their sequences give rise to these properties—or adopt those properties of macrochromosomes—as expected if physical features of microchromosomes give rise to these properties. Genomic assessments of these fused regions within and across falcon species, and Falconidae at large (some genera of which have retained more typically avian chromosomal arrangements), may therefore provide key insights into the causal mechanisms underlying chromosomal and genomic arrangements in birds. Such advances in our understanding of chromosomal arrangements may also help link many peculiarities of the falcon genome to peculiarities in falcon karyotypes, as rates of NUMT insertions (Pereira & Baker, 2004) and indels (Yan, Yi, Sun, Qu, & Yang, 2014) have been shown to vary between micro‐ and macrochromosomes.

### 2.4 The unknowns of falcon genomics

While the genomes of falcons are relatively well characterized for a nonmodel organism, there is still much more work to be done on falcon genomics. Most falcon species have not been sequenced, and those that have been lack supporting resequencing data from other conspecifics. This is compounded by a lack of genome‐wide sequence data from any other members of Falconidae, and a resulting lack of broader evolutionary contexts for existing falcon genomes.

This deficit of genomic data from across *Falco* and other closely related genera obfuscates the evolutionary and ecological factors that have shaped the falcon genome. It is also unclear how peculiarities of falcon genomes can be generalized to the entire group or are species‐specific. In fact, outside of chromosome number, little is known as to whether any of these traits are typical of falcons in general or simply the larger falcons that have been sequenced. The lack of genomes from close relatives of sequenced falcons also means that more detailed analyses of the two existing falcon genomic assemblies have largely been conducted in comparison with Galliformes (i.e., chickens, *Gallus gallus domesticus*, and turkeys, *Meleagris gallopavo*) and the passerine zebra finch, *Taeniopygia guttata* (Zhan et al., 2013). Sampling from across the major clades of falcons (i.e., hobbies, kestrels, and larger falcons) could provide much more detailed insights into the selective forces that have shaped the genomes of falcons during adaptation to specific hunting strategies.

Lack of genome‐wide resequencing data for existing falcon genomes (peregrine, gyr, and saker) greatly restricts estimates of genomic variation within these species and forces the extrapolation of particular genomic traits across them. Estimates of genetic variation in falcons are also currently restricted to heterozygous sites. To put this in perspective, the total number of polymorphic sites was ten times the average number of heterozygous sites per an individual in a resequencing study of turkeys (*M. gallopavo*; Aslam et al., 2012), suggesting that standing genetic variation may be underestimated by an order of magnitude in falcons.

## 3 GENOMICS AND FALCON DIVERSITY AS A RESEARCH SYSTEM

### 3.1 Diversity, divergence and recent speciation

Genome‐wide approaches are at their greatest potential when applied to diverse and recently diverged organisms (Pyron, 2015), and their application to falcons has the potential to provide new insights into speciation ranging from the incipient to the ongoing to the later stages of reproductive isolation. The emergent theory on speciation suggests that speciation can occur through divergent selective forces in the presence of gene flow, a process known as ecological speciation (Schluter, 2009). Vicariance may also drive diversification in nonadaptive contexts by imposing reproductive barriers (Gittenberger, 1991). The degree to which this process has given rise to the diversity of falcons is uncertain: Bayesian reconstructions of biogeographic ranges support adaptive evolution (particularly to migration and habitat type) as the primary drivers of speciation in the genus *Falco* and suggest a relatively limited role for vicariance as a driver of diversity (Fuchs et al., 2015). However, these analyses relied on sequences from select loci, rather than whole‐genome approaches, and have not quantified the extent of gene flow during speciation events. Genome‐wide approaches can be used to reconstruct historic patterns of gene flow, assess whether speciation events occurred during conditions of allopatry, peripatry, or sympatry, and evaluate the finer‐scale mechanics of speciation within falcon species, including novel genomic innovations that may have driven diversification events (Berner & Salzburger, 2015; Gavrilets & Losos, 2009). Several areas remain unresolved with regard to how reproductive isolation evolves in response to divergent selective pressures, including the types of traits that drive divergence, the number of loci and strength of selection underlying this divergence (e.g., strong vs. multifarious selection), and the factors that determine how far organisms progress along the speciation continuum (Nosil, Harmon, & Seehausen, 2009). The recent species‐rich radiations of falcons provide an excellent opportunity to evaluate these hypotheses and assess the specific types of traits that tend to be under the strongest divergent selection, and the commonality of hybrids within captive breeding programs for falconry provides an excellent opportunity to assess how interactions between genes may restrict gene flow between falcon species.

### 3.2 Intraspecific gene flow, local adaptation, and extraordinary geographic range

Falcons also provide many opportunities for studies on local adaptation and gene flow within species. Falcons have one of the broadest distributions of any bird genus (Gill & Donsker, 2019): They are found on all continents except Antarctica and across islands in all of the world's oceans. Occupying a range of 84,425,277 km^2^ (Gaston, Davies, Gascoigne, & Williamson, 2005), peregrine falcons in particular have the broadest species range of any bird (Holroyd & Bird, 2012): These are divided into nineteen recognized subspecies characterized by generally distinct geographic and morphological differences (White, Cade, & Enderson, 2013). These subspecies have the potential to locally adapt to their endemic prey and conditions and can display very marked differences in phenotypes. The smallest subspecies *Falco peregrinus minor*, found in Africa, for example, is less than half the size of the largest subspecies *Falco peregrinus pealei*, which is found in the northwest regions of North America (Jenkins, 1995; Johansson, Linder, Hardin, & White, 1998). Behavioral difference can also be pronounced: Some subspecies are migratory, whereas others are not and differences in nesting habits, breeding seasons, and prey choice have also been documented between subspecies (see White, Cade, et al., 2013 for a thorough review of differences between peregrine subspecies). Similarly marked regional differences also occur among conspecifics of many other widely distributed falcon species such as the American kestrel, *Falco sparverius* (Layne & Smith, 1992; Miller, Mullins, Parrish, Walters, & Haig, 2012; Pearlstine & Thompson, 2004). Detailed genomics studies on such conspecifics have the potential to reveal how gene flow and minor variations in selection regimes can create and maintain diversity within species. Such studies may be of broad interest as they have the potential to reveal the evolutionary mechanisms that drive many commonly observed biogeographic patterns. For example, falcons size distributions appear to be influenced by Bergmann's rule (Meiri & Dayan, 2003), and broadly dispersed falcon species also often display variation in plumage color, which has been linked to Gloger's rule (Moseikin & Ellis, 2004; Olsen, 1982; White, Sonsthagen, et al., 2013), another commonly observed biogeographic principal that states that birds in more humid environments display darker plumage color (Delhey, 2019). The evolutionary forces that drive Bergmann's rule are disputed (McNab, 2010) and Gloger's rule currently lacks a robust evolutionary explanation, although linkage between pigmentation and pathogen resistance has been hypothesized (Delhey, 2019). Intraspecific genomic studies on broadly distributed falcons have the potential to provide new insights into these processes and other similar ecogeographic patterns by revealing specific loci that are under selection during these processes.

## 4 ANTHROPOGENIC IMPACTS ON THE FALCON GENOME AND RESEARCH OPPORTUNITIES

### 4.1 Falconry: An ancient cultural heritage of contemporary economic consequence

Falcons occupy a special position at the interface between anthropogenic and wild systems; it is from this position that their unique potential for use in genomics research emerges. There are few other wild species that share such an intimate, age‐old, and complex relationship with human culture, and no cultural practices so central to this relationship as falconry. While the origins of falconry are hotly debated (Epstein, 1943; Negro, 2018), there is reasonably strong evidence that falconry has been practiced in the Middle East for at least 5,000 years (Canby, 2002). This timeline suggests that falcon husbandry is more recent than that of pigeons (~10,000 BC; Shapiro & Domyan, 2013) and chickens (~6,000 BC; Tixier‐Boichard, Bed'hom, & Rognon, 2011), but older than that of turkeys (300 BC–100 AD; Thornton et al., 2012). However, unlike any of these birds, and despite long‐term use by humans, falcons lack clearly domesticated varieties. This is probably because falconry has traditionally utilized wild‐caught birds or chicks taken from nests. Although earlier sporadic captive breeding of falcons has likely occurred, the first documented captive breeding of falcons occurred in 1939 (Kennedy, 1986) and captive breeding of falcons did not become common until the 1970s (Cade, 1986). Human use of falcons may, however, have had long‐term influences on the genomes of falcons. There is evidence that contemporary falconry has altered the genetic stock of falcons through admixture between escaped falconry birds and native falcons (Rodríguez, Siverio, Siverio, & Rodríguez, 2019). Falcons have been subject to widespread movement and trade by humans for centuries (Gorobets & Kovalchuk, 2017; Tyrberg, 2002), and may have been released by, and escaped, from falconers in sufficient numbers to allow for historic interbreeding between wild birds and those used in falconry as well.

Long‐term use of falcons by humans also provides a peculiar research opportunity: Falcons were commonly kept as pets and sacred animals by ancient Egyptians and were regularly mummified (Morgan & McGovern‐Hoffman, 2008); more recent falcon remains can also be found at archeological sites across medieval Europe (Gorobets & Kovalchuk, 2017; Tyrberg, 2002; Zinoviev, 2016). Both types of samples should provide accessible ancient DNA that can be applied to genomic analyses (Cooper et al., 2001; Mitchell et al., 2014; Welch et al., 2012), and we note that sequencing of ancient DNA from mummified crocodilians has been effectively used to reveal their historical ranges and the long‐term impacts of humans on these (Hekkala et al., 2011). Application of genomic sequencing of ancient falcon DNA could likewise allow for novel insights into historic patterns of range and dispersal among falcons.

Though large‐scale captive breeding of falcons for falconry has only recently become common (Fleming et al., 2011), the evolutionary ramifications of these programs are unknown and could be significant. First, data from other systems suggest that decades of breeding in captivity would create relatively strong selection regimes, even if these are not imposed intentionally (Agnvall, Jöngren, Strandberg, & Jensen, 2012; Christie, Marine, French, & Blouin, 2012; Lacy, Alaks, & Walsh, 2013; Trut et al., 2009): Differential capacity to cope with proximity to humans, thrive and breed under the unnatural conditions of captivity, and freedom from many constraints imposed by natural environments are likely to take their toll (Zeder, 2012). Falcons are also, of course, selectively bred for certain traits that make them more appealing to falconers (e.g., size, particular plumage morphs, pursuit patterns) and often hybridized with one another to obtain these traits (Fleming et al., 2011). Falcons from long‐term hybrid lineages, such as the 7/8 gyrfalcon × 1/8 saker falcon mixes commonly used in the United Arab Emirates, are among the standard stocks of falcons produced and sold. Genomic approaches are needed to document the changes that these practices have brought about in captive lineages. The purity of captive falcon lineages is also in doubt, given the commonality of artificial hybridization; genomic approaches can thus be used to assess these and to detect the role of hybridization in providing genetic material for the production of desired captive lineages. While captive falcons are, at most, within the early stages of domestication, substantial changes have been produced under artificial selection regimes in chickens along similarly short time scales (Tixier‐Boichard et al., 2011). To date, the genomics of domestication remain unstudied in raptors, and studies on the ongoing adaptation and adoption of falcons into human captivity may provide insights into the process of domestication in a totally new group of organisms.

### 4.2 Conservation successes and genomic repercussions

Captive breeding for falconry went on to form the basis for captive breeding for conservation. The depletion of many falcon populations in the 20th century, and their subsequent recovery through captive breeding and release make falcons the epitome of modern conservation success (Abbitt & Scott, 2001; Donlan et al., 2006; Holroyd & Bird, 2012). Despite this distinction, the overall ecological and evolutionary impacts that anthropogenic stressors and specific conservation strategies have had on falcons remain poorly resolved. Falcons have been differentially affected by organochlorides depending on their species and geographic range (Table 2). Exposure to organochlorides was a nearly universal global phenomenon among falcons in the 20th century, but the level of accumulation was strongly influenced by proximity to agriculture and the biomagnification of these pesticides and their metabolites into higher trophic levels (Behrooz, Esmaili‐Sari, Ghasempouri, Bahramifar, & Covaci, 2009), causing bird‐eating falcons to be most affected. While conservation and monitoring efforts were overwhelmingly focused on peregrine falcons, much less is known about the extent of population declines cause by organochlorides in other falcon species. Genomic approaches provide opportunities for the retroactive assessment of population declines and recoveries in other falcon species (Groombridge et al., 2009). Studies on captive quail have suggested that adaptive resistance to organochlorides may be possible in birds (Poonacha et al., 1973), a contention that is further supported to by evidence for interspecific differences in DDT tolerance among falcons (Jarman et al., 1996). As such, genomic approaches can be used to determine the selective pressures and resultant adaptations that organochlorides (and conservation efforts) may have exerted and induced across intra‐ and interspecific falcon populations.

**Table 2:** Pesticide exposure and conservation status of falcon species and populations

Falcon	IUCN	Organochloride effect	Region(s)	Citation(s)
Grey Kestrel	LC	Deleterious	Nigeria	Koeman et al. (1978)
Dickinson's Kestrel	LC	NA	NA	
Sooty Falcon	VU	NA	NA	
African Hobby	LC	NA	NA	
Eleonora's Falcon	LC	Exposure	Aegean	Ristow, Conrad, Wink, and Wink (1980)
	—	Deleterious	Morocco	Clark and Peakall (1977)
Australian Hobby	LC	Thinning	Australia	Olsen, Fuller, and Marples (1982)
Oriental Hobby	LC	NA	NA	
Eurasian Hobby	LC	Deleterious	Spain	van Drooge, Mateo, Vives, Cardiel, and Guitart (2008)
		Exposure	Iran	Behrooz et al. (2009)
Lanner Falcon	LC	Exposure	Sicily	Movalli, Valvo, Pereira, and Osborn (2008)
		Deleterious	Zimbabwe	Tannock, Howells, and Phelps (1983)
		Exposure	South Africa	Smith and Bouwman (2000)
		Deleterious	Kenya	Frank, Jackson, Cooper, and French, (1977)
		Reintroduction	Israel	Bahat (2001)
Saker Falcon	EN	NA	NA	
Red‐necked Falcon	LC	Exposure	Pakistan	Abbasi et al. (2016)
Taita Falcon	VU	NA	NA	
Grey Falcon	VU	Thinning	Australia	Olsen et al. (1982)
Laggar Falcon	NT	NA	NA	
Prairie Falcon	LC	Deleterious	USA	Jarman et al. (2002)
		Exposure	Canada	Jarman et al. (2002)
		Deleterious	USA	Enderson and Berger (1970)
Barbary Falcon	NA	NA	NA	
Peregrine Falcon	LC	Thinning	Australia	Olsen et al. (1982)
		Decline	Australia	Olsen and Olsen (1979)
		Deleterious	Spain	van Drooge et al. (2008)
		Deleterious	Zimbabwe	Tannock et al. (1983)
		Deleterious	Zimbabwe	Hartley, Newton, and Robertson, (1995)
		Deleterious	Scotland	Mearns and Newton (1988)
		Decline	England	Horne and Fielding (2002)
		Decline	Britain	Crick and Ratcliffe (1995)
		None	South America	McNutt et al. (1988)
		Reintroduced	Southern Canada	Holroyd and Bird (2012)
		Reintroduced	Eastern USA	Holroyd and Bird (2012)
		Decline	Western USA	Enderson, Heinrich, Kiff, and White (1995)
		Decline	Arctic North America	Enderson et al. (1995)
		Decline	Subarctic North America	Enderson et al. (1995)
		Deleterious	Greenland	Falk, Møller, and Mattox (2006)
		Reintroduction	Scandinavia	Jacobsen, Nesje, Bachmann, and Lifjeld (2008)
		Deleterious	Australia	Olsen et al. (1982)
		Decline	Russia	Quinn and Kokorev (2000)
Gyrfalcon	LC	Exposed	Norway	Gjershaug, Kålås, Nygård, Herzke, and Folkestad (2008)
		Exposed	Canada	Poole and Bromley (1988)
		Exposed	Iceland	Ólafsdóttir, Petersen, Thordardottir, and Johannesson (1995)
		Exposed	Europe	Koskimies (2005)
Black Falcon	LC	Exposed	Australia	Olsen et al. (1993)
Eurasian Merlin	LC	Thinning	Norway	Gjershaug et al. (2008)
		Thinning	Britain	Newton and Haas (1988)
		Decline	Britain	Bibby and Nattrass (1986)
		Exposure	Iran	Behrooz et al. (2009)
American Merlin	LC	Exposure	USA	Becker and Sieg (1987)
		Deleterious	Canada	Fox and Donald (1980)
American Kestrel	LC	Deleterious	USA	Lincer (1975)
Fox Kestrel	LC	NA	NA	
Seychelles Kestrel	VU	Decline	Seychelles	Groombridge et al. (2009)
Nankeen Kestrel	LC	NA	NA	
Spotted Kestrel	LC	NA	NA	
Lesser Kestrel	LC	Exposure	Spain	Van Drooge et al. (2008)
		Exposure	Spain	Negro, Donázar, Hiraldo, Hernández, and Fernández (1993)
Malagasy Kestrel	LC	NA	NA	
Mauritius Kestrel	EN	Reintroduction	Mauritius	Jones et al. (1995)
Greater Kestrel	LC	Exposure	South Africa	Smith and Bouwman (2000)
Rock Kestrel	NA	NA	NA	
Common Kestrel	LC	Exposure	Spain	Van Drooge et al. (2008)
		Exposure	Pakistan	Abbasi et al. (2016)
		Exposure	Iran	Behrooz et al. (2009)
Banded Kestrel	LC	NA	NA	
Amur Falcon	LC	NA	NA	
Red‐footed Falcon	NT	Exposure	Russia	Henny, Galushin, Khokhlov, Malovichko, and Iljukh (2003)
Orange‐breasted Falcon	NT	NA	NA	
Bat Falcon	LC	Decline	Mexico	Kiff and Peakall (1986)
Aplomado Falcon	LC	Decline	Mexico	Kiff and Peakall (1980)
		Exposure	Mexico	Mora et al. (2011)
New Zealand Falcon	NT	NA	NA	
Brown Falcon	LC	NA	NA	

Note

None—no pesticides detected or no evidence for harm due to pesticides.

Exposure—detection in or strong evidence for the bioaccumulation of pesticides.

Thinning—documented thinning of eggs due to pesticides.

Deleterious—evidence for egg losses or limited population declines due to pesticides.

Decline—evidence for effects of pesticides sufficient to cause a significant population decline.

Reintroduction—population restored by release of moved or captive‐bred birds.

Conservation‐oriented responses to population declines have also varied widely across populations, both within and between falcon species: While peregrine falcons were reintroduced to much of North America and parts of Europe by the movement and captive breeding of birds from other populations (Donlan et al., 2006), many affected peregrine and almost all affected nonperegrine falcon populations appear to have recovered naturally. Several studies have suggested that reintroduction campaigns mitigated losses of genetic diversity in the regions in which they occurred (Brown et al., 2007; Johnson et al., 2010; Ponnikas, Ollila, & Kvist, 2017). However, studies comparing reintroduced populations to naturally recovered populations, which may retain high heterozygosity (Bounas et al., 2018), are lacking, as are studies assessing potential losses in local and locally adaptive genetic diversity among reintroduced and naturally recovered populations. As the legacy of falcon conservation continues to guide similar contemporary reintroduction and management programs (Carroll et al., 2015; Watson, 2018), such studies will help inform ongoing conservation efforts for other species.

### 4.3 Human–falcon interaction in the modern era

In an era of increasing conflict between humans and wildlife, conservation‐oriented studies on falcon genomics may also help answer much more basic questions relating to how animals evolve in relation to anthropogenic habitat alterations (Cooke, Hogan, Isaac, Weaving, & White, 2018). Peregrine falcons naturally nest in cliffs, but have only recently, in the last few decades, begin to commonly inhabit the high rises of cities (Holroyd & Bird, 2012; Luniak, 2004). These urban falcons are now quite common in North America and Europe, and their acclimation to city life has fueled a further range expansion for this already ubiquitous species (Banks et al., 2010). These new habitats represent a natural exploitation of new anthropogenic niche by *F. peregrinus* looking for limited nesting locations and prey (Cooke et al., 2018). The newly colonized environments of city falcons likely represent a dramatic shift in selection regimes from the isolated cliffs of their ancestors. Genome‐wide studies can be used to catalog how these falcons are evolving to these urban environments. Human‐induced habitat alterations represent one of the greatest ongoing threats to biodiversity. Studies on how animals adapt to these are of considerable and growing interest but have to date primarily focused on mammals and invertebrates (Alberti, 2015; Hulme‐Beaman, Dobney, Cucchi, & Searle, 2016; McDonnell & Hahs, 2015). Falcons, and peregrine falcons in particular, afford an opportunity to study response to urbanization in a raptor, and in the context of their already complex conservation history, exemplify the need for genomic studies on the evolutionary responses of other animals to humans.

Genomic approaches may also reveal hidden links between falconry, urbanization, and the falcon conservation programs of the late 20th century. City falcons are suggested to have arisen in part as an unintentional consequence of captive breeding and release (Fleming et al., 2011). As peregrine falcons seem to choose nesting locations more similar to the nesting locations in which they were reared (Faccio et al., 2013), captive‐released birds may have imprinted on human‐made structures and gained a preference for these after their release. If this explanation holds true, it would represent an astounding real‐world example of heritable nongenetic alterations in the ecology of an animal and highlight the role that such processes can have in providing the phenotypic plasticity for adaptation to human habitats. While genomic approaches cannot directly confirm this process, they could be used to indirectly test this hypothesis by assessing the ultimate origins of city falcons as decedents of either native or captive‐bred progenitors, potentially on the basis of nonlocal ancestry (Tordoff & Redig, 2001).

## 5 FALCON GENOMICS AND THE MICROBIOME

The close association between humans and falcons also extends to their associated communities of symbionts (parasites, commensals, and mutualists), and genomic approaches offer an opportunity to study these as well (Jovel et al., 2016; Weinstock, 2012). Studies on cross‐transmission between humans and falcons are of immediate applied concern as falcons can carry and (directly or indirectly) transmit several medically relevant pathogens to humans, including avian influenza virus (Naguib et al., 2015), West Nile virus (Busquets et al., 2012), and Newcastle disease virus (Samour, 2014). All of these viruses can also transmit to falcons through ingestion of infected prey, making falcons likely reservoir hosts. Falcons may also act as important reservoirs for disease dispersal due to the natural long‐distance migration of many falcons and the human‐facilitated movement of falcons resulting from falconry. The cross‐border movement of falconry falcons has, notably, been implicated in the transmission of highly pathogenic H5N1 avian influenza virus into the Middle East, Europe, and Africa, following ingestion of infected prey in central Asia (Naguib et al., 2015). Captive falcons are also subject to novel pathogens. Opportunistic *Aspergillus* spp. are of particular concern among captive falcons (Beernaert, Pasmans, Waeyenberghe, Haesebrouck, & Martel, 2010; Tarello, 2011); bacterial communities of falcons have also been linked to various health problems that are associated with the failure of some falcons to thrive in captivity and the hatching success of falcon eggs (Peralta‐Sánchez et al., 2018). Metagenomic approaches can be applied to better understand the environmental and genetic factors driving differences in susceptibility to these organisms. At present, nothing is known about the typical healthy microbiome of falcons, but falconers have turned to probiotic supplements to give their falcons an edge in competitions. Genomic characterization of falcon microbiomes has the potential to produce better products for falconers and improve veterinary care for falcons by establishing a healthy baseline for microbiome comparisons. The competitive aspects of falconry also provide an opportunity for the study of the microbiome in relation to well‐established metrics of performance across several closely related species. Falcon microbiomes are also of interest at a more basic level; the recent radiation of falcons opens the door to studies on how microbiomes speciate with their hosts. At present, little is known about this subject, but a recent and well‐cited study on great apes reports mixed patterns of cospeciation and cross‐species colonization in hominids (Moeller et al., 2016). Falcons present an opportunity to study this idea across a much wider array of species and hybrids, in the presence of variable and recurrent gene flow, and across many more climates and habitats.

## 6 CONCLUSIONS

Decreasing sequencing costs and new genomic technologies are creating new possibilities for population‐scale and genome‐wide sequencing of nonmodel organisms (Ellegren, 2014; van Nimwegen et al., 2016): Falcons possess a combination of traits that makes them exceptional candidates for expanded sequencing efforts. From a practical perspective, their small genomes and use in falconry facilitate efficient study (Zhan et al., 2013). More broadly, their significant and recently arisen interspecific diversity provides excellent opportunities for the study of evolution and speciation in a bird (Fuchs et al., 2015), just as the broad geographic ranges (Gaston et al., 2005; Holroyd & Bird, 2012) and the considerable intraspecific diversity of their constituents makes them excellent candidates for fine‐scale studies on gene flow and local adaptation (White, Cade, et al., 2013). Their suitability for assessment of basic scientific questions is coupled with a long history of complex relationships with humans (Negro, 2018) that add to their cultural and economic importance and make them exemplary candidates for genome‐wide studies.

## CONFLICT OF INTEREST

The authors have no competing interests to declare.

## AUTHOR CONTRIBUTIONS

Justin J. S. Wilcox performed the primary background research and writing for this review. Stéphane Boissinot and Youssef Idaghdour provided vital expertise and guidance for this review, and made major contributions to its presentation and content.

## Data Availability

Data have not been archived because this article does not contain data.
